# Effect of Avastin on the number and structure of tumor blood vessels of nude mice with A549 lung adenocarcinoma

**DOI:** 10.3892/etm.2014.1991

**Published:** 2014-09-24

**Authors:** NALI ZHANG, GUOJUN ZHANG, YOUGUANG ZHENG, TONGBING WANG, HONGLEI WANG

**Affiliations:** 1 Department of Respiratory Medicine, The First Affiliated Hospital of Zhengzhou University, Zhengzhou, Henan 450003, P.R. China; 2 Department of Respiratory Medicine, Luoyang Central Hospital Affiliated to Zhengzhou University, Luoyang, Henan 471009, P.R. China; 3 Department of Cardiovascular Medicine, The First Affiliated Hospital of Henan University of Science and Technology, Luoyang, Henan 471003, P.R. China

**Keywords:** Avastin, A549 lung adenocarcinoma cells, vessel number, vessel structure, antitumor

## Abstract

The aim of the present study was to investigate the effect of Avastin on the number and structure of tumor blood vessels of nude mice with A549 lung adenocarcinoma. A total of 30 nude mice were randomly divided into three groups, namely the control, the Avastin I (Avastin 3 mg/kg) and the Avastin II (Avastin 6 mg/kg) groups. Following treatment, ELISA was used to detect the expression level of vascular endothelial growth factor (VEGF) in tumor tissues. The microvascular density in tumor tissues and tumor vascular pericyte coverage was detected by immunofluorescence. The tumor growth and survival rate of mice in the three groups were also analyzed. Compared with the control group, the Avastin I and II groups exhibited significantly decreased VEGF levels and microvascular density in the tumor tissues, with the decrease in the Avastin II group being more prominent (P<0.05). After 7 days of treatment, the vascular pericyte coverage in the tumor tissues of mice in the Avastin I and II groups was significantly increased compared with that in the control group mice (P<0.05). Compared with the control group, the mice in the Avastin I and II groups exhibited a significantly decreased tumor growth rate and this effect was dose-dependent. The survival rate of mice in the Avastin I and II groups was significantly increased compared with that of the mice in the control group (P<0.05). In conclusion, Avastin significantly decreased the microvascular density of the tumor in nude mice with A549 lung adenocarcinoma and also significantly increased tumor vascular pericyte coverage, inhibited tumor growth and increased the survival rate of the mice, through its potent antiangiogenic activity.

## Introduction

Tumor vessels lay the foundation for tumor growth and metastasis; therefore, the antitumor treatment focusing on tumor angiogenesis has become a focus in clinical tumor treatment (1,2). It was previously reported that, in addition to the significant neovascularization, the tumor blood vessels are characterized by abnormal vascular structure, including multiple branches, structural disorder, overlapping of endothelial cells and lack of pericytes (3). Therefore, the treatment targeted at tumor vessels is aimed at reducing their number and also improving their abnormal structure. Vascular endothelial growth factor (VEGF) induces the proliferation and migration of endothelial cells and increases microvascular permeability (4–6). It was previously demonstrated that the expression level of VEGF in tumor tissues is significantly increased and promotes tumor angiogenesis (7,8). Avastin is a novel anti-VEGF humanized monoclonal antibody and is able to combine with all VEGF isomers with high affinity, indirectly inhibiting VEGF from binding to its receptor, which is the mechanism through which Avastin exerts its biological effects (9,10).

In 2001, Jain proposed the theory of vascular normalization and considered that vascular normalization concurrently with the administration of agents targeting tumor blood vessels may conduce to the antitumor effects of treatment (11). Avastin was shown to inhibit tumor angiogenesis *in vivo* and *in vitro* and is currently the main drug used for targeting the blood vessels of malignant tumors (12). However, the number of studies on whether Avastin facilitates the normalization of the tumor vasculature is limited. Therefore, in this study, A549 lung adenocarcinoma cells were used to construct a nude mouse model to analyze the antitumor effect of Avastin and its effect on tumor vessel number and structure, in order to add to the theoretical basis for antitumor treatment with Avastin.

## Materials and methods

### Model construction and treatment

A total of 30 BALB/c nude mice were purchased from Shanghai Laboratory Animal Center of the Chinese Academy of Sciences, Shanghai, China. The mice were aged 6–8 weeks and weighed 19–22 g. The animals were maintained under specific pathogen-free conditions. This study was performed in strict accordance with the recommendations of the Guide for the Care and Use of Laboratory Animals of the National Institutes of Health. The animal use protocol was reviewed and approved by the Institutional Animal Care and Use Committee of the First Affiliated Hospital of Zhengzhou University.

A549 cells were cultured in RPMI-1640 culture medium supplemented with 10% fetal bovine serum (FBS; HyClone Laboratories, Logan, UT, USA) until the exponential growth phase. The culture medium was discarded and the flask was washed with twice with phosphate-buffered saline (PBS), followed by the addition of 0.25% pancreatic enzymes (Gibco, Grand Island, NY, USA) to digest the cells. Subsequently, RPMI-1640 with 10% FBS was added to stop the digestion and the cells were centrifuged at 1,500 × g for 3 min. The cell sediment was rinsed twice with PBS and resuspended in PBS, with the cell concentration regulated to 5×10^7^/ml. The cells (5×10^6^/100 μl) were inoculated in a fat pad near the axilla in the left rib of each nude mouse. After 1 week, when the tumors had grown to 100–150 mm^3^, the tumor-bearing nude mice were randomly divided into three groups (n=10 per group) as follows: The control group (each mouse was intraperitoneally injected with 100 μl PBS every other day, for a total of eight times); the Avastin I group [Avastin (Roche, Basel, Switzerland) was intraperitoneally injected at a dose of 3 mg/kg every other day, for a total of eight times]; and the Avastin II group (Avastin was intraperitoneally injected at a dose of 6 mg/kg every other day, for a total of eight times). Starting from the first day of treatment, the tumor size was measured daily.

### Survival rate analysis

After the mice model was constructed successfully, when the tumors had grown to 100–150 mm^3^, the tumor-bearing nude mice were randomly divided into three groups (n=10 per group) as follows: The control group (each mouse was intraperitoneally injected with 100 μl PBS every other day, for a total of 8 times); the Avastin I group [Avastin (Roche, Basel, Switzerland) was intraperitoneally injected at a dose of 3 mg/kg every other day, for a total of 8 times]; and the Avastin II group (Avastin was intraperitoneally injected at a dose of 6 mg/kg every other day, for a total of 8 times). Starting from the first day of treatment, the survival rate of the mice was observed during treatment for 25 days.

### ELISA

After 7 days of treatment, the mice in the three groups were euthanized by cervical dislocation, and 0.1-g tumor tissue samples were placed in radioimmunoprecipitation assay buffer for 10 min to prepare the tissue lysate. Subsequently, a protease inhibitor was added. The liquid was placed on ice for 30 min and then centrifuged at 15,000 × g for 10 min. The supernatant fluid was collected and the VEGF level in the tumor tissues was determined with the VEGF ELISA kit (R&D Systems Inc., Minneapolis, MN, USA) according to the manufacturer’s instructions.

### Immunofluorescence method

Tumor tissues were embedded in optimal cutting temperature compound to prepare frozen sections, which were fixed by paraformaldehyde and sealed with 10% goat serum. Rat anti-mouse CD31 (0610017-C) and rabbit anti-mouse α-smooth muscle actin (SMA; CSB-E09343h) antibodies (Abcam, Cambridge, UK) were proportionally diluted and dripped onto the tissue sections, coated and incubated overnight 4°C. On the following day, the sections were rinsed with PBS three times. Fluorescein isothiocyanate (FITC) goat anti-rat (BA1101) and phycoerythrin goat anti-rabbit fluorescent secondary antibodies (BA1034; Boster Biological Technology, Ltd., Wuhan, China) were proportionally diluted and dripped onto the tissue slides, incubated at 37°C for 1 h and rinsed with PBS three times. Subsequently, DAPI was used for nuclear staining, the slides were covered with cover slips and observed under a fluorescence microscope (Olympus, Tokyo, Japan) at ×400 magnification. A total of 10 visual fields were randomly selected among the tumor sections from each group to calculate the vessel number. The proportion of normal blood vessels was calculated as follows: 40 blood vessels were randomly selected (magnification, ×400), the blood vessels stained red by CD31 were counted and the proportion of FITC α-SMA-positive vessels was calculated.

### Statistical analysis

All the data were analyzed with SPSS 17.0 statistical software (SPSS Inc., Chicago, IL, USA) and measured data were expressed as means ± standard deviation. Multi-group comparisons were conducted with one-factor analysis of variance and inter-group comparison was performed with the least significant difference method. Comparisons of survival rates were performed with the Kaplan-Meier method and the log-rank test. P<0.05 was considered to indicate a statistically significant difference.

## Results

### Avastin inhibits the expression of VEGF in tumor tissues

**Figure 1:**
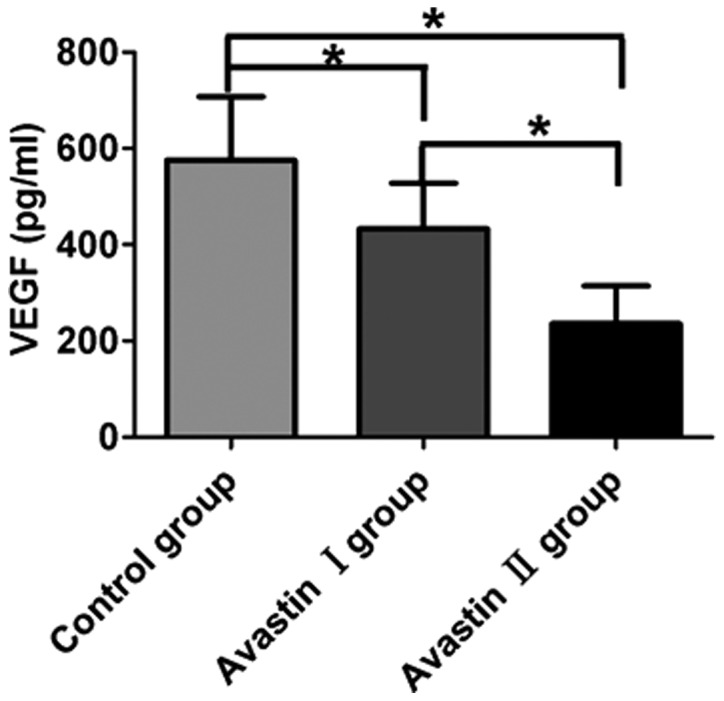
Comparison of vascular endothelial growth factor (VEGF) protein levels in the tumor tissues from the three groups. ^*^P<0.05.

Compared with the control group (575.72±49.75 pg/ml), the Avastin I (433.32±49.75 pg/ml) and Avastin II (235.75±40.17 pg/ml) groups exhibited significantly downregulated VEGF protein levels in the mouse tumor tissues and the differences were statistically significant (P<0.05). Following treatment, the Avastin II group exhibited a more prominent downregulation of VEGF expression compared with the Avastin I group and the difference was statistically significant (P<0.05) (Fig. 1).

### Avastin controls the tumor blood vessel number and promotes vascular pericyte coverage

**Figure 2:**
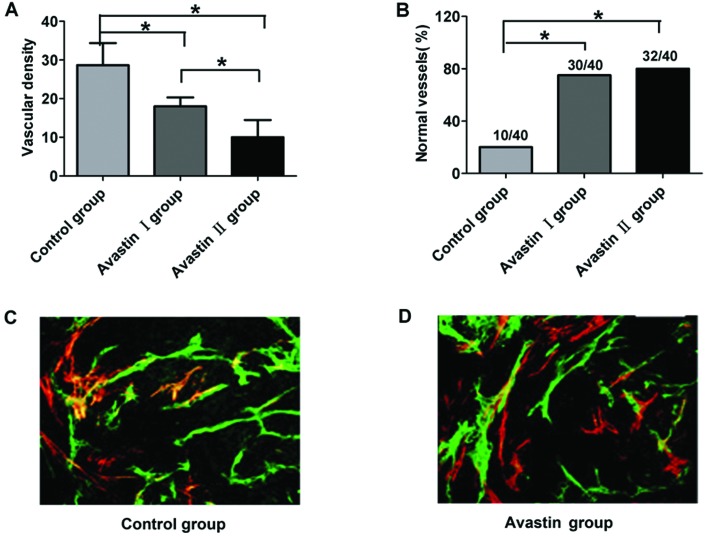
Comparison of (A) tumor vessel number and (B) tumor vessel normalization persentage among the three groups after 7 days of treatment;^*^P<0.05. Tumor vessel structures (C) in the control group and (D) in the Avastin group by immunofluorescence.

After 7 days of treatment, the Avastin I and II groups exhibited markedly decreased vascular density compared with the control group, while the vascular density of the Avastin II group decreased more significantly (P<0.05) (Fig. 2A). Compared with the control group, the proportion of normal vascular structures in the mouse tumors from the Avastin I and II groups was distinctly increased (P<0.05). There was no statistical significance in the proportion of normal vascular structures between the Avastin I and II groups (P>0.05) (Fig. 2B–D).

### Avastin inhibits tumor growth and increases the survival rate of tumor-bearing mice

**Figure 3:**
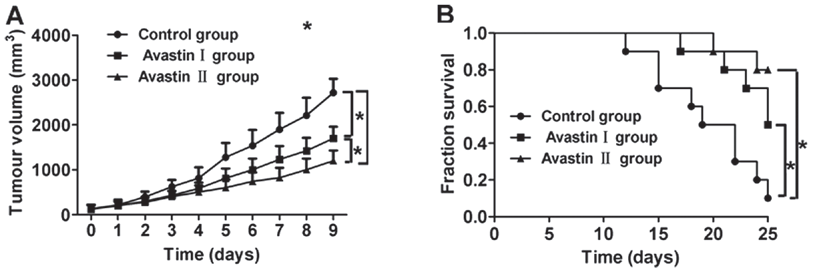
Comparison of (A) tumor growth and (B) survival among the three groups. ^*^P<0.05.

Compared with the control group, the tumor growth in mice from the Avastin I and II groups slowed down significantly after treatment, while the growth rate of the tumors in the Avastin II group was significantly lower compared with that in the Avastin I group, with a statistically significant difference (P<0.05) (Fig. 3A). Furthermore, the survival rates of the mice in the Avastin I and II groups were significantly higher compared with that of control group (P<0.05) (Fig. 3A). There was no statistically significant difference in survival rate between the Avastin I and II groups (P>0.05) (Fig. 3B).

## Discussion

Folkman (13) reported that when a solid tumor grows to 1–2 mm, tumor growth depends on the oxygen and nutrients supplied by newly formed blood vessels, whereas the time to tumor metastasis is also associated with tumor angiogenesis. Therefore, tumor blood vessels are essential for tumor growth, infiltration and metastasis and antitumor treatment targeting tumor vessels has become a focus of investigation. Several antiangiogenic drugs are currently used in clinical practice and it was demonstrated that they are able to significantly inhibit tumor angiogenesis and, thus, control tumor growth (14,15). Of note, in addition to the prominent angiogenesis, the tumor vasculature is characterized by severe structural and functional abnormalities. The abnormal vessel structure hinders the delivery of antitumor drugs to the tumor tissues, compromising their antitumor effect. Accordingly, treatments targeting tumor vessels must be aimed at inhibiting tumor angiogenesis and also at normalizing the existing vessels, in order to promote antitumor drug delivery to solid tumors via vascular system.

VEGF plays an important role in tumor angiogenesis and blocking of VEGF signaling may effectively inhibit tumor angiogenesis (16). Avastin is a novel anti-VEGF humanized monoclonal antibody, which is able to significantly inhibit tumor angiogenesis *in vivo* and tumor growth (17). Our results demonstrated that Avastin at a concentration of 3 mg/kg significantly downregulated VEGF levels in tumor tissues and inhibited angiogenesis in A549 lung cancer tissues. Jain (11) observed an abnormal lack of pericyte coverage in the tumor vasculature and this observation may indirectly reflect the structural changes of the tumor vessels (18,19). In our experiment, we demonstrated that after 7 days of treatment with Avastin, the of tumor vessel pericyte coverage was significantly increased compared with the control, suggesting that Avastin promotes the normalization of the tumor vasculature. Our result were consistent with those previously reported (20).

In our study, Avastin inhibited tumor angiogenesis and promoted the normalization of tumor vessels, significantly inhibiting the growth of A549 lung tumors in treated mice in a dose-dependent manner. The antitumor effects of Avastin are mediated through the inhibition of tumor angiogenesis and also through the normalization of the tumor vasculature, which increases the amount of Avastin delivered to the tumor tissues to exert its antiangiogenic effect. Avastin also significantly increased the survival rate of A459 tumor-bearing mice.

In conclusion, Avastin significantly inhibits tumor angiogenesis and promotes normalization of tumor vessels, thus inhibiting tumor growth and increasing survival rate. However, the mechanism underlying the vascular normalization achieved by Avastin requires further investigation.
